# Identifying Target Genes for Engineered Genetic Incompatibility in Fish

**DOI:** 10.1007/s10126-026-10617-2

**Published:** 2026-04-25

**Authors:** Leland Feist, Samuel E Erickson, Nicholas Jacob, Isabel Ameli, Carolyn Malecha, Julie Badger, Michael J Smanski

**Affiliations:** 1 Biotechnology Institute, University of Minnesota, Saint Paul, MN 55108 USA; 2 Department of Biochemistry, Molecular Biology, and Biophysics, University of Minnesota, Saint Paul, MN 55108 USA; 3 Conservation Science Graduate Program, Saint Paul, MN 55108 USA

**Keywords:** Engineered genetic incompatibility, Non-persistent genetic biocontrol, Common carp, Zebrafish

## Abstract

Genetic biocontrol approaches promise to complement existing physical and chemical methods as part of integrated pest management (IPM) strategies for the control of aquatic invasive species (AIS). Engineered Genetic Incompatibility (EGI) is a strategy for producing organisms that are reproductively isolated from wild conspecifics and could be used as non-persistent genetic biocontrol agents. Previously successfully demonstrated in yeast and insects, here we report early-stage research and development results towards translating this approach from insects into fish. Using *Danio rerio* (zebrafish) as a model system, we report the identification of target genes and sequence-programmable transcriptional activators (PTAs) that are suitable for EGI development in fish and evaluate their performance *in vivo*, in a model organism. We also describe several challenges faced when integrating the component parts into a complete system capable of displaying complete genetic incompatibility with wild-type conspecifics. Lastly, we discuss the steps needed to translate EGI from a model fish species to a target invasive species such as common carp (*Cyprinus carpio*).

**Supplementary Information:**

The online version contains supplementary material available at 10.1007/s10126-026-10617-2.

## Introduction

**Fig. 1:**
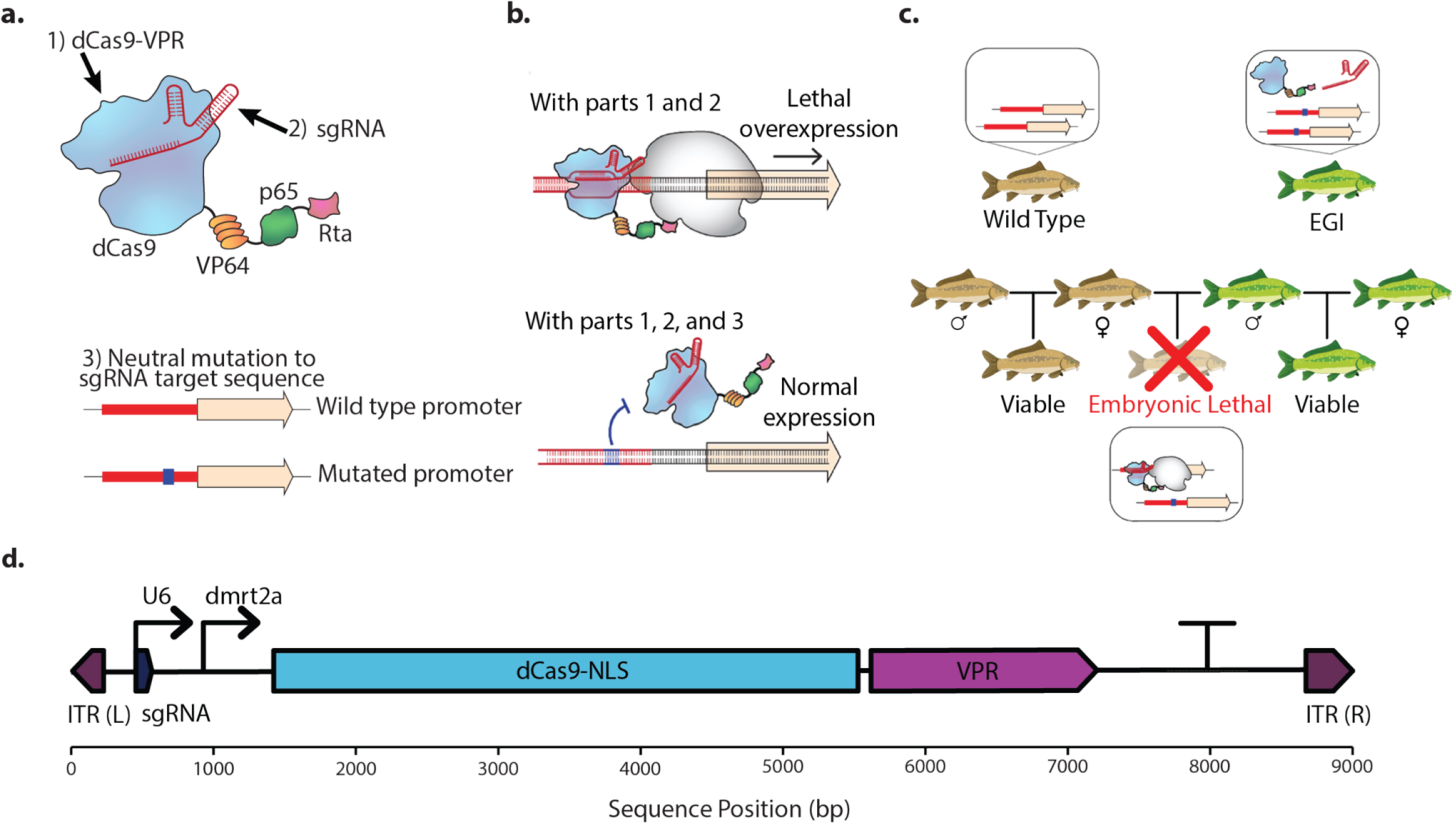
Overview of the EGI mechanism. **(a)** The three components of EGI technology: A CRISPR-based programmable transcription activator (PTA), a single-stranded guide RNA (sgRNA), and the introduction of a resistance mutation in the promoter sequence matching the sgRNA. **(b)** Effect of the PTA and sgRNA on gene expression in the presence and absence of a mutated promoter sequence. **(c)** Hybrid lethality only occurs in the offspring of an EGI and wild-type cross. EGI can be crossed with EGI to produce viable EGI offspring. **(d)** Plasmid map of PTA and sgRNA driven by the *dmrt2a* and *U6-1* promoter, respectively. The flanking inverted terminal repeats (ITRs) are required for integration into the host genome via the Tol2 transposase. Figure adapted from Maselko et al. (2020)

Aquatic invasive species present unique challenges for population control due to their high fecundity and increased difficulty to treat due to their aquatic environments (Havel et al. 2015; Thomaz et al. 2014). Traditional management strategies, such as netting, trapping, closing locks and dams, and piscicides, are not species-specific and cannot achieve population suppression alone. To overcome these challenges, genetic biocontrol technologies are being developed to be used alongside traditional methods as part of integrated pest management (IPM) strategies, resulting in effective species-specific suppression (Hubert et al. 2019).

Genetic biocontrol strategies use rationally engineered agents to spread genes into wild populations via sexual reproduction. These genes can result in suppression of the wild population or replacement of the wild population with a genotype that dampens or eliminates the deleterious traits of the pest. While there are diverse genetic biocontrol strategies, they share common strengths, including their species-specificity and their compatibility with IPM strategies (Alphey 2014; Esvelt et al. 2014). One subcategory of approaches genetically modifies the pest organism such that they cannot successfully reproduce with the original, unmodified population. Wild-type conspecifics that mate with these agents will not have offspring capable of surviving to adulthood.

According to the *biological species concept* brought forward by the zoologist Mayr in 1942, a species is defined as "groups of actually or potentially interbreeding natural populations which are reproductively isolated from other such groups" (Mayr 1999; Aldhebiani 2018). Engineered Genetic Incompatibility (EGI) generates a biological speciation event, resulting in post-zygotic reproductive isolation. Hybrid lethality is achieved by coupling a dominant lethal transgene (e.g., only one copy is required for the lethal effect) with a recessive resistance allele (e.g., a single copy of the resistance allele is insufficient for resistance). This allows the EGI genotype, which is homozygous for each of these components, to reproduce successfully with other EGI individuals while being incompatible with wild-type strains that lack resistance alleles (Maselko et al. 2020).

Demonstrated examples of EGI to achieve hybrid lethality through a three-part mechanism is shown in Fig. 1. A CRISPR-based programmable transcriptional activator (PTA) drives lethal ectopic or over-expression of a target gene. The PTA is composed of a dCas9 (a catalytically inactive Cas9) that has been fused to transcriptional activating domains. For example, the dCas9-VPR PTA uses domains from VP64, p65, and Rta to drive target gene transcription (Chavez et al. 2015). A single guide RNA (sgRNA) is responsible for directing the PTA to the wild-type promoter sequence of the target gene. Once bound, the PTA recruits transcriptional machinery, thus up-regulating the expression of the target gene and resulting in lethality of hybrid offspring (Maselko et al. 2020; Zinselmeier et al. 2024).

PTA-driven lethality in the parent EGI strain is avoided by introducing a mutation to the sequence targeted by the sgRNA, thus preventing the sgRNA-PTA complex from binding. Individuals that are homozygous for the PTA, sgRNA, and mutated promoter (hereafter called the "EGI genotype") are resistant to ectopic expression and are fully fecund when mated to compatible EGI genotypes. However, hybrids between EGI and wild-type are hemizygous for each component, and the sgRNA-PTA complex can drive lethal ectopic expression from the single copy of the wild-type target promoter. In short, EGI utilizes a haplosufficient lethality mechanism coupled with a haploinsufficient resistance allele to achieve selective lethality and genetic incompatibility.

EGI could be leveraged in multiple ways to combat invasive species. One strength of EGI is the ability to block gene flow between genetically modified and wild-type populations. EGI could be used as a form of sterile/incompatible male release, similar to what has been used on a large scale to combat populations of screwworms, tsetse flies, medflies and mosquitoes (Leftwich et al. 2015; Dowell et al. 2007; United States Department of Agriculture 2014; Spinner et al. 2022; Plá et al. 2021). The genetic incompatibility is "hard-wired" into the genetics of the EGI agents, and these agents can be propagated by conventional means. This differentiates EGI from genetic biocontrol with conventional sterile insect technique, where batch sterilization via irradiation can negatively affect lifespan and mating competitiveness (Hooper 1972; Langley et al. 1974). Unlike unidirectional cytoplasmic incompatibility methods, EGI is less sensitive to release of female control agents (Sinkins 2004). Similar to all sterile/incompatible release strategies, EGI is self-limiting and inherently confinable, which contrasts with many RNA-guided gene drives that have a high persistence due to their super-Mendelian inheritance patterns (Esvelt et al. 2014).

EGI technology has previously been demonstrated in the model eukaryote *Saccharomyces cerevisiae* and the model insect *Drosophila melanogaster* (Maselko et al. 2017, 2020). The developmental biology of fish and insects is substantially different. As an intermediate step on the pathway towards developing EGI for applications in AIS, we sought to characterize the required components in a model fish species (Badger et al. 2025). The importance of developing self-limiting genetic pest management strategies is demonstrated by recent advancements of a similar technology in zebrafish, highlighting the feasibility of EGI for invasive species management (Tizard et al. 2023). Here, we independently characterize the components of EGI in zebrafish, *Danio rerio*, including the identification of a lethal target gene, the design and validation of sgRNAs, and the integration of the PTA. Finally, we analyzed the resulting hybrid lethality of this EGI mechanism *in vivo* and describe the potential breeding schema to efficiently develop EGI in a target non-model organism.

## Materials and Methods

### Fish Husbandry and Rearing

Zebrafish were reared at the University of Minnesota Zebrafish Core Facility. Zebrafish were kept at 28°C with a photoperiod of 14 hours of light and 10 hours of dark. Conventional husbandry protocols were followed.

Embryos were kept in "embryo water" for the first 7 days following fertilization. Embryo water was made by adding 3 grams of Instant Ocean, purchased from Spectrum Brands (Middleton, Wisconsin), to 50 L of MilliQ water.

To induce spawning, a single male and a single female were placed on opposite sides of a spawning tank with a removable divider and left overnight. The divider was removed as the lights were turned on at 8:30 am to induce spawning. Immediately following fertilization, the embryos were collected and the adult fish were returned to their original tanks. Zebrafish were given at least one week of time between spawning events.

When required, zebrafish were anesthetized in a tank containing 0.017% MS-222 and euthanized via cold water bath. All experiments involving animals were carried out with the approval of the Institutional Animal Care and Use Committee (IACUC protocol ID: 2203-39903A).

**Fig. 2:**
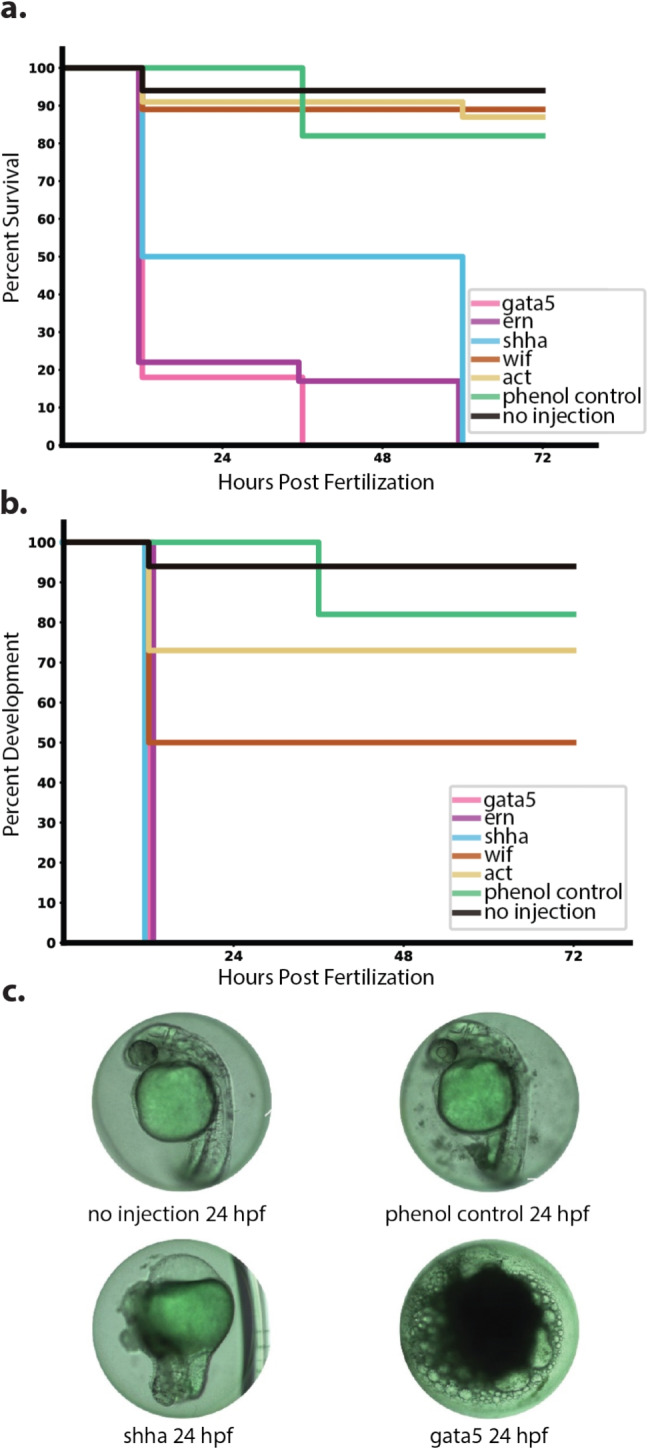
Identification of lethal target genes. **(a)** Survival curve of zebrafish embryos microinjected with the mRNA of various genes which were potentially lethal when over-expressed. **(b)** Developmental curve showing percent of unhealthy embryos determined by the absence of vital body structures required for survival. Embryos were injected with 3 nL containing 1,000 pg of the target gene mRNA and 400 pg of green fluorescent protein (GFP) RNA. **(c)** From left to right, top to bottom, representative images of embryos 24 hours post fertilization receiving no injection, injected with phenol red dye, *shha* mRNA, and *gata5* mRNA. Graphs show averages of three replicates of 100 embryos, (n=300)

### Plasmid Creation

Annotated plasmid sequences are available on Benchling (San Francisco, California) with access links listed in Supplementary Table 1 along with their descriptions. The zebrafish *U6-1* promoter was used to express the sgRNA. The zebrafish *dmrt2a* promoter was used to express the PTA. The zebrafish $\alpha$*-crystallin* promoter was used to express mCerulean in the eye of transgenic individuals. Unless otherwise noted, all enzymes were purchased from New England Biolabs. Promoters were amplified from the zebrafish genome via PCR. Plasmids were constructed using PCR and type IIs restriction digestion ligation reactions and grown in *Escherichia coli* DH5*α* which was purchased from New England Biolabs (Ipswich, Massachusetts) (Engler and Marillonnet 2014). PCR was performed using Q5 DNA polymerase. *E. coli* was transformed via heat shock. PCR and heat shock were performed using the C1000 Touch Thermal Cycler purchased from Bio-Rad (Hercules, California). *E. coli* was grown at 37°C in BD Difco LB Broth Lennox purchased from Fisher Scientific (Waltham, Massachusetts) with 50 mg/L kanamycin antibiotic. Plasmid purification was performed using the QIAprep Spin Miniprep Kit purchased from Qiagen (Hilden, Germany). Plasmids were validated by PCR screening and Sanger sequencing.

### Identification of Lethal Gene Targets via mRNA Microinjection of Embryos

cDNA encoding the *gata5*, *ern*, *shha*, *wif*, and *act* genes was purchased from TransOMIC Technologies (Huntsville, Alabama) and cloned into the pTRI-Xef vector for *in vitro* synthesis of 7-methylguanosine-capped mRNA using the Ambion mMESSAGE mMACHINE RNA synthesis kit from Invitrogen (Waltham, Massachusetts). mRNA was quantified via NanoDrop spectrophotometry using the Nanodrop 2000c spectrophotometer purchased from Thermo Fisher Scientific (Waltham, Massachusetts) and RiboGreen analysis following lithium chloride precipitation.

Next, 1,000 pg of the *in vitro* transcribed mRNA was microinjected into 100 single-cell stage embryos for each of the five target genes. For each microinjection, a 3 nL droplet was used containing 1,000 pg of target gene mRNA and 400 pg of green fluorescent protein (GFP) mRNA as a marker of successful mRNA delivery and translation. Each droplet contained phenol red to act as an injection marker and track the injected embryos. A no-injection control and a phenol red only control were included as well. After 4–6 hours, the green fluorescent embryos were separated and divided into individual wells on a 96-well plate with embryo water. These embryos were observed for deformities and death daily for three days. The microinjector used was the PLI100 air-based microinjector from Harvard Apparatus (Holliston, Massachusetts). Glass capillaries (1B100F-4; World Precision Instruments; Sarasota, Florida) were pulled using the Model P-87 Needle Puller from Sutter Instrument (Novato, California) to make microinjection needles. The microscope used to view zebrafish embryos was the Nikon SMZ1500 (Tokyo, Japan).

**Fig. 3:**
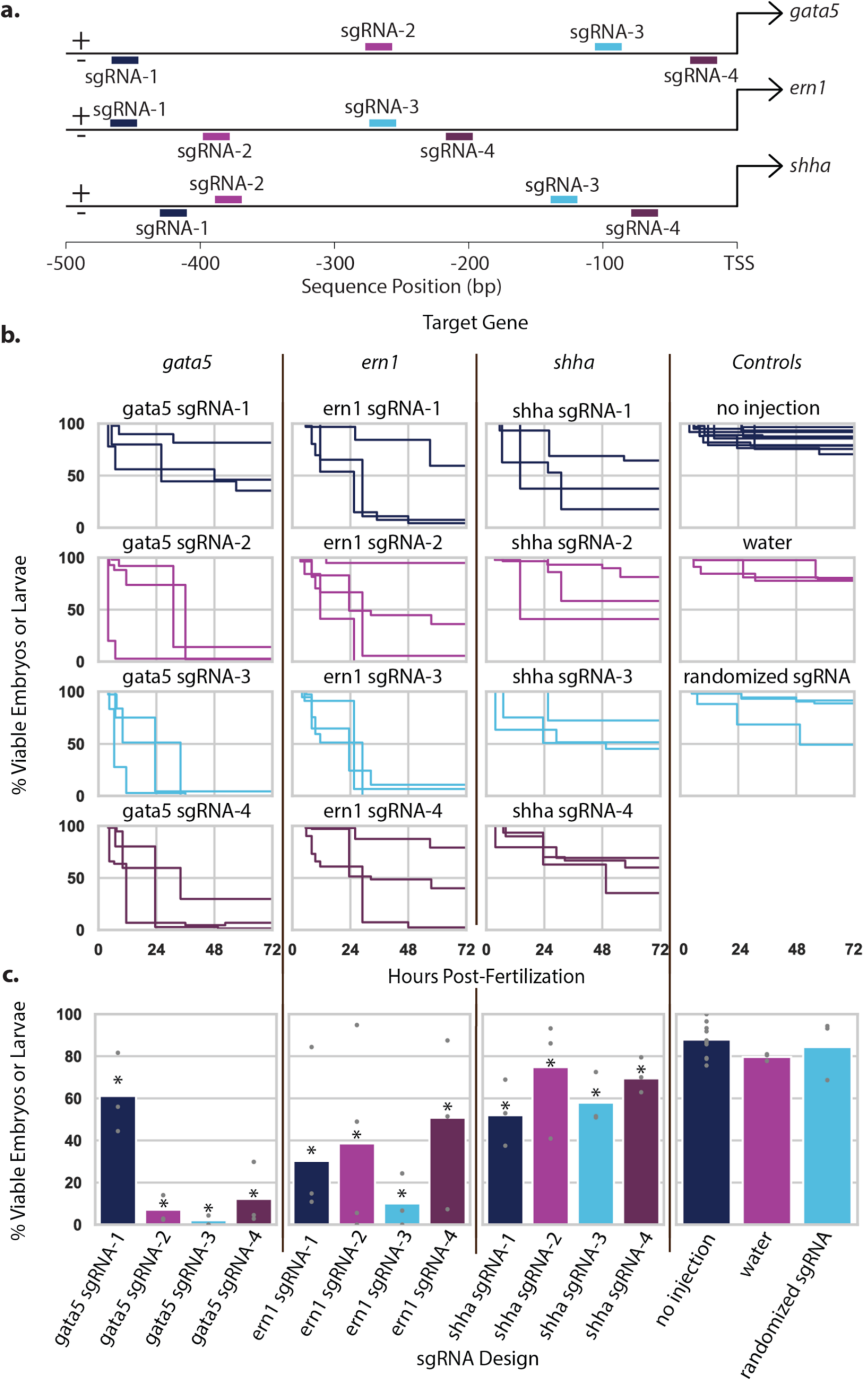
Identifying suitable sgRNAs. **(a)** Target location of each tested sgRNA. **(b)** Survival curves of zebrafish embryos that received no injection, an injection of water, or a construct containing the programmable transcriptional activator (PTA) and different sgRNAs. **(c)** The percent survival of embryos one day post fertilization. The sample size for each data point ranged from 119–438. Note that the bar chart shows overall percent viability from three replicates and the data points show individual percent viability from each replicate. Because of differences in sample sizes, the bar is not the arithmetic mean of the data points. A chi-squared analysis was performed, an asterisk denotes a significant difference in lethality compared to the no injection control $P \le 0.01.$

### Designing and Testing Suitable Single-stranded Guide RNAs

Four sgRNAs were generated for each of the target genes using gene sequences from the GRCz11 *D. rerio* reference genome from NCBI (Bethesda, Maryland). Suitable binding sites within 500 bp upstream of the transcriptional start site were identified and designed with the Benchling CRISPR design tool. The top four sgRNA binding site candidates were chosen for each gene. sgRNAs matching these binding sites were then cloned into plasmids and used for microinjections.

pT3TS-Tol2 was a gift from Stephen Ekker (Addgene plasmid # 31831 ; http://n2t.net/addgene:31831 ; RRID:Addgene_31831) (Balciunas et al. 2006). The Tol2 transposase encoding plasmid pT3TS-Tol2 was linearized via *Sap*I enzymatic digest (Balciunas et al. 2006). This linearized template was then transcribed *in vitro* using the Invitrogen mMESSAGE mMACHINE T7 Transcription Kit and purified by lithium chloride precipitation and redissolved in 25 µL of RNase-Free water. Zebrafish embryos at the single cell stage were microinjected and received an injection of 1 nL containing 20 ng/µL of the corresponding plasmid and 20 ng/µL of mRNA encoding Tol2 transposase and 20 ng/µL of GFP mRNA using the same microinjection setup described above. To act as controls, a plasmid containing a sgRNA with a random sequence was injected, along with injections of water. Additionally, many control zygotes were not injected. Following injection, all dead, deformed, or unhealthy embryos were removed each day for three days. Unhealthy was defined as an embryo that one could reasonably expect to not mature or reproduce but that appeared to still be alive. Following data collection, a chi-squared test of independence was performed.

**Fig. 4:**
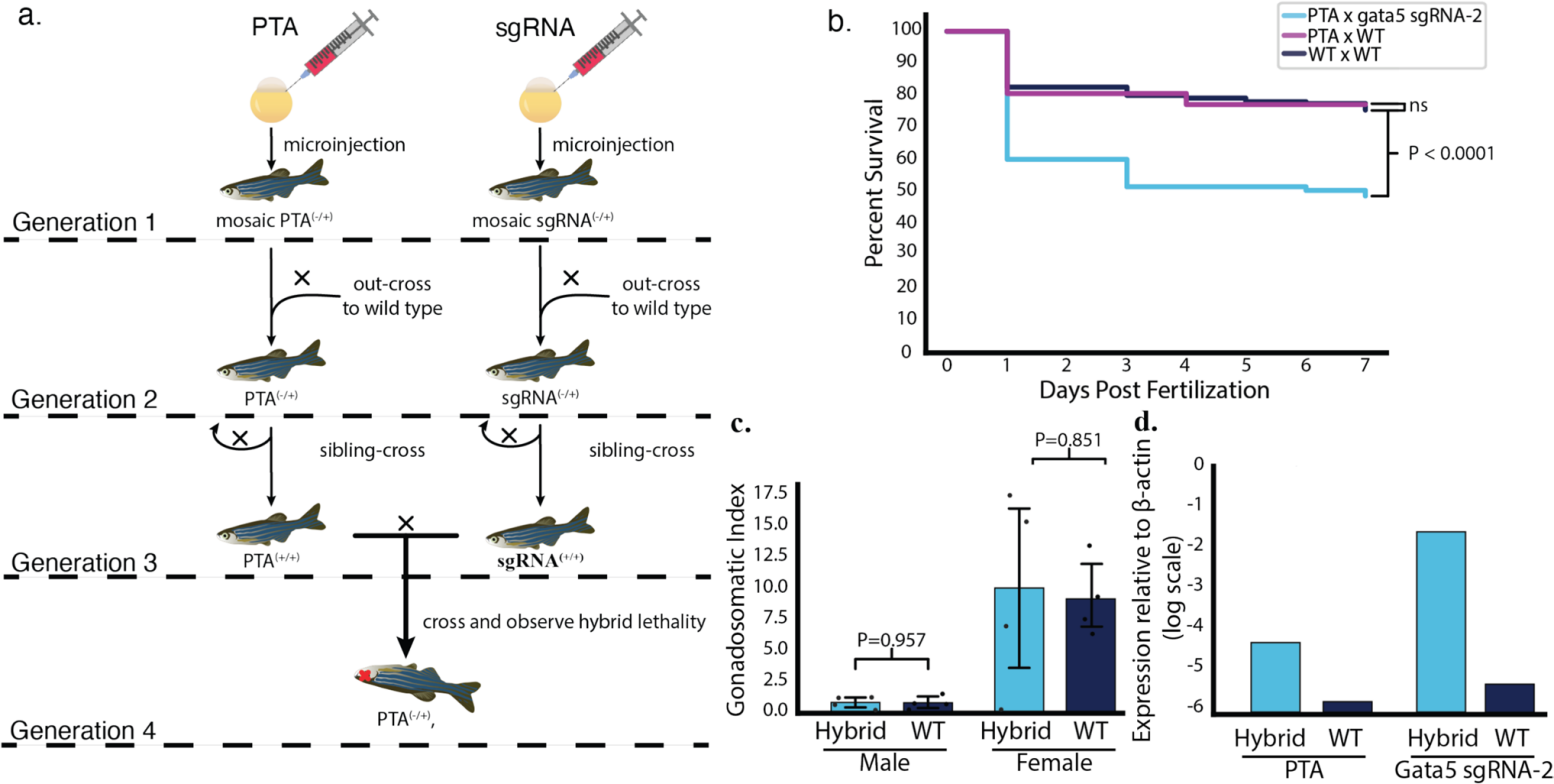
Breeding scheme, survival curve of zebrafish from PTA x *gata5* sgRNA-2 cross, and subsequent development. **(a)** The breeding method followed to insert and test the lethality of different PTA x sgRNA pairs. The PTA and sgRNA were integrated via the Tol2 transposase. Hybrid lethality is expected to be observed in the fourth generation if ectopic or over-expression of the target gene is sufficient to result in lethality. **(b)** Percent survival resulting from the cross of a homozygous PTA and homozygous *gata5* sgRNA-2 zebrafish (blue), the cross of the same homozygous PTA zebrafish and a wild-type zebrafish (purple), and the cross of two wild-type zebrafish (grey), (n=200 for each cross). **(c)** The gonadosomatic index of the male and female surviving PTA x *gata5* sgRNA-2 offspring compared to wild-type of the same age (n=16). **(d)** Fold change in expression of the PTA and *gata5* sgRNA-2 in the PTA-*gata5*-sgRNA-2 x wild-type offspring and wild type relative to $\beta$-*actin*. Data is presented as $log_{2}$ fold changed normalized to $\beta$-*actin* and calculated using the ${\Delta \Delta Cq}$ method. Bars represent the mean of 3 technical replicates (n=1)

### Characterizing Hybrid Lethality

Two stable zebrafish lines were created that express either the PTA or sgRNA. The breeding schema to generate transgenic homozygous individuals is described in Fig. 4a. The PTA or sgRNA were integrated into the zebrafish genome by Tol2 transposase activity following the microinjection protocol described above. Each embryo received an injection of 1 µL containing 20 ng/µL of plasmid and 20 ng/µL of Tol2 mRNA during the single cell stage within 20 minutes after fertilization. Successful transgenesis was confirmed by expression of mCerulean in the eye driven by the $\alpha$-*crystallin* promoter. The mosaic transgenic individuals resulting from these microinjections were then crossed with a wild-type zebrafish to produce hemizygous transgenic fish, which were then crossed with siblings to generate roughly 25% homozygous offspring. Homozygous individuals were confirmed by crossing them with wild-type zebrafish and counting the number of transgenic offspring, indicated by the expression of mCerulean in the eye. If all of their offspring expressed mCerulean in the eye, the parent was deemed homozygous for the transgene.

Once identified, a homozygous PTA zebrafish was crossed with a homozygous sgRNA zebrafish. To act as a control for PTA toxicity, the same homozygous PTA zebrafish was crossed with a wild-type zebrafish a week later. Two wild-type zebrafish were crossed to act as a control as well. For analysis, 100 embryos were selected at random from each clutch just after fertilization. Each day for seven days, the dead embryos were removed, a 100% water change was performed, and the surviving embryos were counted (Fig. 4b). This process was repeated, for a total of 200 embryos and two spawning events per pair of zebrafish. Following data collection, Kaplan-Meier curves were generated and a log-rank test was performed.

The surviving PTA x sgRNA heterozygotes were allowed to grow for four months to reach sexual maturity. Four males and four females from the PTA x *gata5* sgRNA-2 cross were randomly selected and crossed with wild-type zebrafish. These crosses were repeated twice for a total of 48 attempted spawning events among 16 breeding pairs. The number of eggs laid per cross was recorded on day 0. Dead embryos were removed and 100% water changes were performed daily. Six days after fertilization, the number of surviving fry were counted. After crossing the heterozygotes, dissection and extraction of the gonads was performed following the protocol outlined by Gupta and Mullins (2010). The weight of each zebrafish was measured prior to dissection. Following dissection, the weight of the extracted gonads was measured. The gonadosomatic index was calculated using the following formula:

$$ \text {Gonadosomatic Index} = (M_g / M_t) * 100 $$

where $M_g$ is the mass of the gonads and $M_t$ is the total body mass. Following the calculation of the gonadosomatic index, a Welch’s T-test was performed.

### Reverse Transcription Quantitative PCR of the PTA and sgRNA

Four one-month old PTA x *gata5* sgRNA-2 and four one-month old wild-type zebrafish were euthanized via ice bath and homogenized using a mortar and pestle. mRNA was purified from full body homogenate samples using the TRIzol RNA extraction method (Chomczynski 1993). Primers targeting the $\beta$-*actin* mRNA sequence were used from Tang et al. (2007). Reverse transcription quantitative PCR (RT-qPCR) was performed using the Luna Universal Probe One-Step RT-qPCR Kit purchased from New England Biolabs following the manufacturer protocol. RT-qPCR was performed on the CFX Connect Real-Time PCR Detection System purchased from Bio-Rad. RT-qPCR data was analyzed using the $\Delta \Delta$Cq method.

## Results

### Identification of Lethal Target Genes

We first sought to identify endogenous genes that, when ectopically or over-expressed, were capable of causing lethality. Five candidate genes were selected for their importance in cellular differentiation and embryonic development: *gata binding protein 5* (*gata5*), *endoplasmic reticulum to nucleus signaling 1* (*ern*), *sonic hedgehog signaling molecule A* (*shha*), *wnt inhibitory factor 1* (*wif*), and $\beta$-*actin 1* (*act*). mRNA encoding the candidate gene was injected into zebrafish embryos, with GFP mRNA, vector-only, or uninjected controls included to measure microinjection efficiency, lethality from microinjection, and egg/embryo quality, respectively.

As seen in Fig. 2a, over-expression of three genes resulted in 100% lethality within 72 hours post fertilization: *gata5*, *ern*, and *shha*. The strongest level of lethality was caused by *gata5* over-expression with 82% embryo lethality after 12 hours and 100% lethality after 36 hours. *ern* over-expression resulted in 78% lethality after 36 hours and achieved 100% lethality within 60 hours. Finally, *shha* over-expression resulted in 50% lethality in 12 hours and 100% lethality within 60 hours. *wif* and *act* over-expression failed to result in 100% lethality over 72 hours with *wif* over-expression resulting in only 11% lethality and *act* over-expression resulting in 18% lethality. Embryos that received no injections saw high survival, resulting in only 6% lethality, while embryos injected with phenol red resulted in 18% lethality. A majority of the lethality was observed within the first 12 hours post injection with another large drop in survival 36 hours post injection. Raw data can be found in Supplemental Table 3.

As seen in Fig. 2b, many embryos displayed developmental abnormalities that deviated from normal zebrafish embryo development (Kimmel et al. 1995). Abnormalities, including death, were observed in 100% of the *gata5*, *ern*, and *shha* groups within 12 hours. *wif* over-expression resulted in 50% abnormalities while *act* resulted in 27% abnormalities. Embryos that received no injections resulted in 6% developmental abnormalities while embryos that received injections of phenol red resulted in 18% abnormalities. Representative images of developmental abnormalities can be seen in Fig. 2c. Raw data can be found in Supplementary Table 4. Embryos injected with *shha* mRNA displayed evidence of disruption during the gastrula period of development, lacking a head but possessing a tail bud, whereas embryos injected with *gata5* mRNA lacked any evidence of an organized body plan, instead appearing as an amorphous mass of disorganized cells. Due to the observed high levels of abnormalities and lethality, we selected *gata5*, *ern*, and *shha* as our three target genes for further development of EGI.

### Identification of Suitable sgRNAs

After identifying three promising target genes, we sought to confirm that dCas9-based PTAs could drive sufficient levels of gene expression for lethality. The location at which the PTA binds relative to the transcriptional start site (TSS) can affect the expression level of the target gene (Casas-Mollano et al. 2020). The Benchling CRISPR design tool was used to identify four binding sites within 500 bp upstream of the TSS and four sgRNAs were designed matching each binding site for each of the target genes, shown in Fig. 3a.

A series of 15 constructs (four per gene plus three controls with randomized sgRNA spacer sequences to control for lethality that may occur from a PTA and sgRNA pair searching for a nonexistent binding site) consisting of the PTA and a single sgRNA was created. Expression of the PTA was driven by the *dmrt2a* promoter while expression of the sgRNA was driven by the *U6-1* promoter. We confirmed via amplicon sequencing (data not shown) that the target sequences were present in our lab brood of zebrafish. Each construct was integrated into the genome of recently fertilized zebrafish embryos via the Tol2 transposase (Suster et al. 2009).

As seen in Fig. 3, the percent of viable embryos after 72 hours varied between each sgRNA, further supporting that the location where the PTA binds relative to the TSS affects the expression of the target gene. At a significance threshold of 0.01, we observed significant differences in the survival of each sgRNA compared to the no-injection group. However, we observed a large amount of variation from gene to gene and sgRNA to sgRNA. The highest level of lethality resulted from the *gata5* sgRNAs. Among three replicates, *gata5* sgRNA-1 had an average viability of 61% and bound 446bp upstream of the TSS. This is much higher than *gata5* sgRNA-2, 3, and 4, which resulted in 7%, 2%, and 12% survival and bound 257 bp, 123 bp, and 69 bp upstream of the TSS, respectively. Targeting *ern1* resulted in the second highest lethality with *ern1* sgRNAs 1, 2, 3, and 4 resulting in 30%, 17%, 10%, and 51% survival and bound 447 bp, 378 bp, 254 bp, and 197 bp upstream, respectively. Finally, *shha* sgRNAs 1, 2, 3, and 4 resulted in 52%, 75%, 58%, and 69% survival and bound 410 bp, 369 bp, 172 bp, and 119 bp upstream, respectively. We observed an average survival of 84% among the three randomized sgRNAs. We found no significant differences between groups that received no injection, received an injection of water, or received an injection of a PTA and a randomized sgRNA sequence. Due to their high levels of lethality, we decided to move forward with sgRNAs 2 and 3 for the *gata5* gene.

### Characterizing Hybrid Lethality

After the identification of suitable sgRNAs, we aimed to analyze the hybrid lethality resulting from inheriting a chromosomally integrated PTA and sgRNA. Three separate zebrafish lines were created. One line contained the dCas9-VPR PTA expressed from the reproductive and nervous system-specific *dmrt2a* promoter (Zhou et al. 2008; del Pozo et al. 2020). The other two lines contained constructs expressing sgRNA-2 or sgRNA-3 targeting *gata5* (Fig. 3), driven by the polymerase III-dependent *U6-1* promoter. Each injected construct also had a fluorescent reporter gene comprising *mCerulean* driven by the $\alpha$-*crystallin* promoter to provide a facile way to confirm transgenesis.

Figure 4a shows the microinjection and breeding scheme used to create zebrafish homozygous for the PTA, gata5 sgRNA-2, or gata5 sgRNA-3. We confirmed homozygosity by outcrossing fish with the fluorescent marker to wild-type and screening the percentage of offspring inheriting the fluorescent marker. Through this method, we identified a single zebrafish homozygous for the PTA and multiple zebrafish homozygous for *gata5* sgRNA-2. However, we were unable to identify an individual homozygous for *gata5* sgRNA-3.

We expected to see increased lethality in the offspring of the PTA and sgRNA-2 cross, resulting from lethal over- or ectopic expression of *gata5*. As seen in Fig. 4b, we observed a mortality rate of 51.5% in the PTA x *gata5* sgRNA-2 offspring (hereafter referred to as ’hybrids’) compared to a mortality rate of 24.5% in the wild-type controls. To account for any lethality that may occur due to the presence of the PTA machinery, the same homozygous PTA zebrafish was also crossed with a wild-type zebrafish. Lethality from this cross was indistinguishable from wild-type. Raw data can be found in Supplementary Table 8.

For control via EGI, viability of hybrids would be tolerable if the surviving hybrids were sterile. To determine whether the surviving hybrid offspring from the PTA x *gata5* sgRNA-2 cross were fertile, we raised them four months to sexual maturity. We then crossed the mature hybrids with wild-type zebrafish and found them to be competent for sexual reproduction, producing offspring that survived at least six days post-fertilization. We observed 13 spawning events for hybrids and found no significant differences in the number of eggs laid, the percentage of eggs hatched, or the number of surviving fry six days post fertilization compared to wild-type (Supplementary Fig. 2). Furthermore, we did not find significant differences in the gonadosomatic index between hybrids and wild-type zebrafish, as seen in Fig. 4c. These results indicate that the surviving offspring of the PTA x *gata5* sgRNA-2 cross possessed functional reproductive systems and were fertile. Raw GSI data and metadata can be found in Supplementary Table 9 and Supplementary Fig. 2.

### Confirming PTA and *gata5* sgRNA-2 Expression via RT-qPCR

To confirm that the PTA and *gata5* sgRNA-2 were expressed in these PTA x *gata5* sgRNA-2 offspring, RT-qPCR was performed (Fig. 4d). $\beta$-*actin* was used as a reference gene for normalizing measurements from each sample. Transcript from the PTA was measured at levels 30-fold greater than background (the amount detected in wild-type, which does not contain the PTA gene, is considered background). The sgRNA component, *gata5* sgRNA-2, was expressed in the hybrids at levels 6000 times greater than the limit of detection in wild-type. Raw data and additional information can be found in Supplementary Tables 6 and 7.

**Fig. 5:**
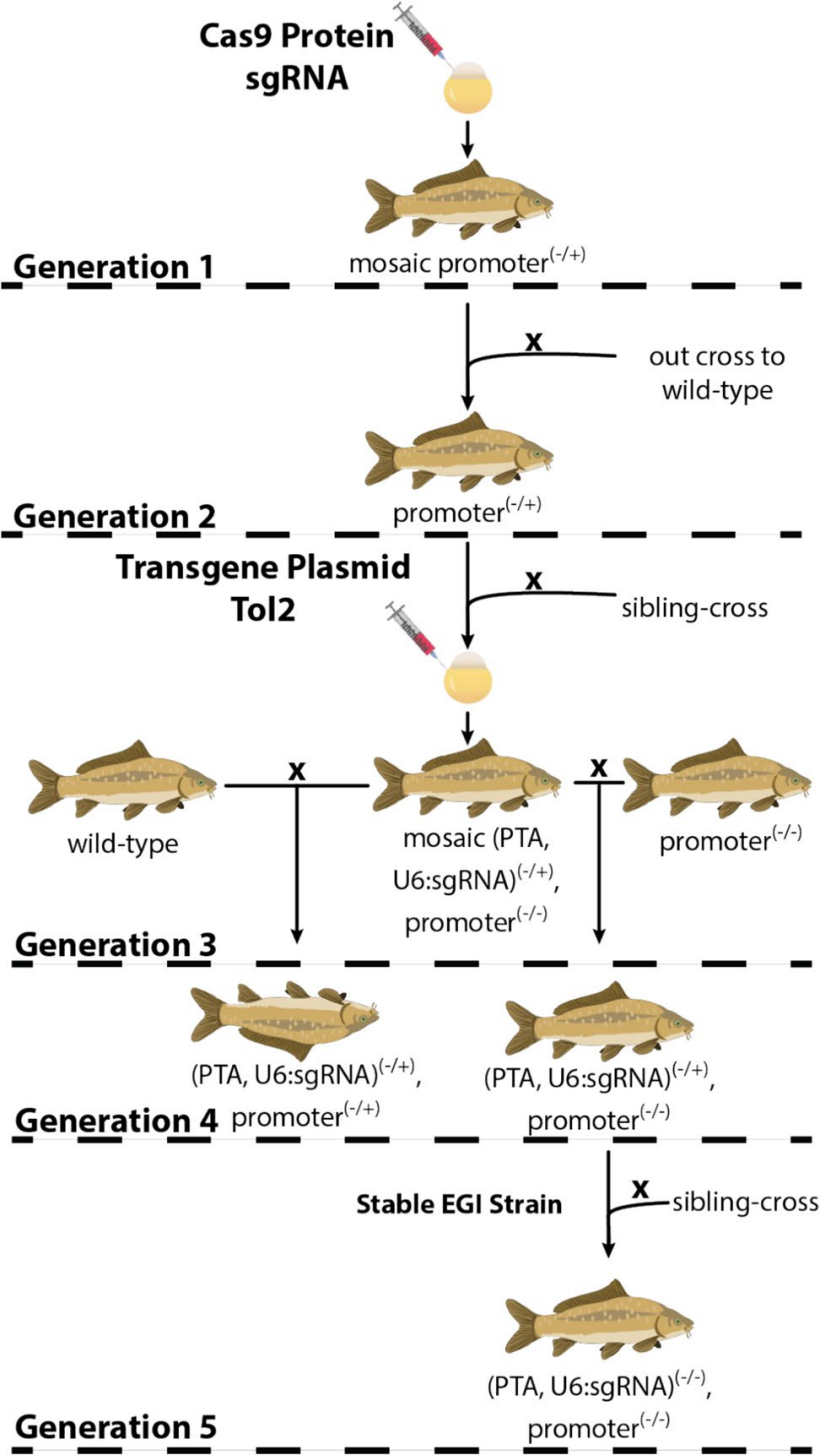
Proposed EGI Carp Breeding Scheme. The proposed breeding scheme to integrate each component of EGI in a non-model invasive species. Target promoter mutations are generated via microinjection with sgRNA and Cas9 protein in the first generation. After stabilizing the via out-crossing to wild type in generation 2, a sibling cross can be performed in generation 3. Mutated promoter homozygotes can be selected via microinjection with the lethal transgene plasmid, which is integrated via the Tol2 transposase. The resulting third generation can be crossed to wild type to screen for lethality, and individuals with sufficient lethality can be crossed to a mutated promoter mutant from generation 3. Finally, two hemizygous EGI genotypes should be crossed to produce the final stable homozygous EGI strain

## Discussion

In this study, we laid the foundation for translating EGI into fish. Three main components are required for EGI: an endogenous lethal target gene (a PTA), a sgRNA pair capable of driving lethal ectopic or over-expression, and a protective mutation in the EGI organism. We began by directly injecting transcribed mRNA *in vitro* into zebrafish embryos to identify genes that would be lethal if over-expressed. Because the level of mRNA abundance introduced through direct injection may not be achievable via PTA-driven expression from the endogenous genetic locus, we next targeted select genes using a dCas9-VPR PTA. Finally, we established whether lethal over- or ectopic-expression was achievable when the PTA components were expressed from the chromosome.

Established EGI systems in *S. cerevisiae* and *D. melanogaster* targeted genes that are essential for cellular integrity e.g., *actin* in yeast) or early development e.g., *pyr*, *wg*, and *upd1* in flies) (Maselko et al. 2017, 2020). Target genes and pathways from these previously published systems were ineffective in fish in our work. Direct injection of $\beta$-*actin* transcript in zebrafish embryos did not drive embryo lethality (Fig. 2). Injecting *wif* transcript, which modulates the *wnt* signaling pathway, was similarly ineffective, despite this pathway being a suitable target in flies. It should be noted that the *wnt*-related target in flies, *wg*, is a positive regulator of signaling, whereas the *wif* gene is a negative regulator of the pathway in fish (Poggi et al. 2018). Previous reports of *wif* over-expression driving severe developmental defects in vertebrates were not seen in this study (Poggi et al. 2018; Dan et al. 2013). It is possible that more complex regulation downstream of gene transcription can temper the phenotypes expected from an overproduction of transcript from $\beta$-*actin* and *wif* in fish. We were successful in identifying three genes capable of causing 100% lethality from over-expression within 72 hours post fertilization in zebrafish: *gata5*, *ern*, and *shha*. The transcription factor *gata5* is responsible for cardiovascular differentiation and has been shown to cause embryonic morphogenesis such as an enlarged heart when over-expressed (ZFIN 2025; Lou et al. 2011). The lethality observed by ectopically expressing *gata5* is likely due to misdirected cell fate, resulting in lethality (Fig. 2). *shha*, a gene that plays a critical role in cell differentiation and survival, caused pericardial edema and limb agenesis at 24 hours post-fertilization (Fig. 2c) (Ingham and Placzek 2006). *ern1* encodes the inositol requiring enzyme 1, a regulator of the unfolded protein response, which can trigger apoptosis. Its over-expression led to 100% lethality within 60 hours post-fertilization (Fig. 2a) (Hetz and Papa 2018). Each of these three genes displayed some level of enhanced lethality when expression was driven by dCas9-VPR. We expressed dCas9-VPR with the *dmrt2a* promoter, which is active in the endocrine, immune, and nervous systems (Zhou et al. 2008; Thisse and Thisse 2005; del Pozo et al. 2020). Because of this, we expect the lethality was due to ectopic expression of these developmental genes, although this was not confirmed experimentally..

The spacing between sgRNA-PTA binding and the TSS can affect the strength of over-expression yet is still unpredictable (Casas-Mollano et al. 2020). It is expected that sgRNAs that bind immediately upstream of the TSS would have the highest positive effect on transcription, and that this positive effect would decrease as the sgRNA moves farther upstream of the TSS (Casas-Mollano et al. 2020; Cheng et al. 2013). We observed the highest lethality from sgRNAs that bound 100–300 bp upstream of the TSS, but this trend was variable between target genes (Fig. 3). While this variability in lethality highlights the importance of sgRNA positioning and gene regulation, additional factors may influence hybrid lethality.

Measuring the expression of *gata5* via RT-qPCR revealed that an injection of the lethal plasmid expressing the PTA and *gata5* sgRNA-2 resulted in a roughly twofold increase in *gata5* transcript 12 and 15 hours post fertilization (Supplementary Fig. 1). It would be expected that expression of the PTA is tissue-specific due to the use of the *dmrt2a* promoter, therefore expression of the PTA is expected to be higher in the target tissues compared to the expression observed from whole-embryo mRNA extraction. Additionally, we observed that the time of death corresponded to the onset of *dmrt2a* expression, suggesting that the mechanism of toxicity matches the EGI design.

Interestingly, *gata5* sgRNA-2 failed to drive the same degree of embryonic lethality observed in Fig. 3 when the PTA components were inherited and expressed from the chromosome versus when it was integrated into the genome via Tol2 mRNA and plasmid microinjection, similar to the results of Tizard et al. (2023). It is likely that many copies of the plasmid were retained after microinjection (i.e., not all of the plasmid was integrated by Tol2), leading to a copy-number difference between the experiments shown in Figs. 3 and 4b. Alternatively, it is possible that epigenetic silencing diminished the expression of the PTA components during the four generations of breeding that were required to perform the experiment in Fig. 4. Interestingly, while we were able to identify an individual homozygous for the PTA and multiple homozygous individuals for *gata5* sgRNA-2, we were unable to identify an individual homozygous for gata5 sgRNA-3. This is possibly due to the construct integrating into the chromosome at a gene that is essential, but this was not confirmed

We predict, based on our group’s experience in demonstrating functional EGI in yeast and flies and the results of Tizard et al., that the hybrid lethality could be improved by tuning the activity of the PTA (Tizard et al. 2023). This could be achieved by replacing the cis-regulatory elements (e.g., promoter, 5’-UTR, etc.) with elements from genes that are more highly expressed than *dmrt2a*. It could also be achieved by creating more PTA transgenic fish that differ in the chromosomal integration locus, as positional effect variegation is known to influence transgene expression levels (Reute and Spierer 1992). Another possible optimization is in PTA design, as several iterations of PTAs have been developed since dCas9-VPR (Casas-Mollano et al. 2023, 2020). However, these optimizations are unlikely to generate knowledge that will directly translate to engineering the system in invasive fish species whose genome architectures are substantially different from zebrafish.

Figure 5 describes a proposed line of experiments for translating EGI into an invasive fish species, using common carp (*Cyprinus carpio*) as an example. The data presented here supports the further development of EGI targeting *gata5* or *ern1* (Fig. 3).

EGI technology has shown to be adaptable to many different organisms (Maselko et al. 2017, 2020; Buchman et al. 2021; Waters et al. 2018; Zinselmeier et al. 2025) and is effective in suppressing target populations in laboratory cage studies (Upadhyay et al. 2022). While EGI has several beneficial qualities for genetic biocontrol, including non-persistence and obviating batch sterilization requirements, it has several weaknesses as well. The engineering required to make an EGI genotype is complex, and generating complete EGI agents may take a decade or more in fish with long generation times (Fig. 5). This could be mitigated by combining it with other genetic biocontrol methods and accelerating its development with technology such as surrogate host technology (Yoshizaki and Yazawa 2019). Additionally, EGI-wild-type hybrids could persist and proliferate if there is genetic diversity in the target population that provides resistance to PTA-driven over-expression e.g., single-nucleotide polymorphisms in the sgRNA binding site (Feltman et al. 2022). Modeling suggested that this genetic resistance can be overcome with multiple mutually-incompatible EGI lines (Sychla et al. 2022).

The severe and persistent impacts of AIS justify the need for effective IPM strategies. EGI is a potential tool that is attractive for IPM strategies due to its self-limiting nature when used in sterile male release programs. Our overall goal in this study was to lay the foundation for translating EGI to target AIS by identifying target genes and PTA designs in a model fish species. Alternative genetic biocontrol methods, such as sex-ratio-biasing techniques or gene drives, offer their own sets of strengths and weaknesses. It is likely that different genetic biocontrol methods will be better suited for deployment in different watersheds. We recommend parallel development of diverse control strategies in controlled environments to maximize the options available to invasive fish control stakeholders (Badger et al. 2025).

## Supplementary Information

Below is the link to the electronic supplementary material.

Supplementary file 1 (pdf 378 KB)

## Data Availability

Plasmid sequences are available on Benchling with access links included in Supplementary Table 1. All raw data and codes used to visualize the data can be found in the Data Repository for U of M (DRUM). (Feist et al. 2025) Access link: https://hdl.handle.net/11299/277308

## Declarations

### Artificial Intelligence Generated Content

The artificial intelligence program ChatGPT, owned by OpenAI (San Francisco, CA), was used to aid in generating the python code used to perform statistical tests and visualize raw data. All AI outputs were edited and corrected as necessary. AI was not used to review the literature, generate text, or generate/interpret data. We maintain that this paper is our own original work. All code and raw data have been made available through the Data Repository for U of M (DRUM) (See Data Availability section for more details).
