# Supplementary material for: Identifying Target Genes for Engineered Genetic Incompatibility in Fish
Source: Mar Biotechnol (NY). 2026 Apr 25;28(3):74. doi: 10.1007/s10126-026-10617-2 (PMC13110222; doi:10.1007/s10126-026-10617-2)
Supplement: Supplementary file 1 — (pdf 378 KB) [file 10126_2026_10617_MOESM1_ESM.pdf]

Supplemental Information

Supplemental Methods

Reverse transcription quantitative PCR of *gata5*. To monitor *gata5* expression in zebrafish embryos at the two-cell stage, embryos were injected with a plasmid containing a sgRNA targeting the *gata5* promoter in combination with the PTA. Embryos were injected with 1.4 nL of solution containing 10 ng/µL plasmid DNA, 20 ng/µL Tol2 transposase mRNA, and nuclease-free water. Control embryos were injected with 1.4 nL nuclease-free water. At 12 and 15 hours post-injection, 60 embryos from each treatment group were collected, flash frozen in liquid nitrogen, and pooled into three biological replicates of 20 embryos per replicate. Frozen samples were homogenized in a microcentrifuge tube using a plastic mortar and pestle. Total RNA was extracted using the NucleoSpin RNA Plus XS Kit purchased from Takara Bio (San Jose, California) according to the manufacturer's instructions. cDNA synthesis was performed using 300 ng of RNA per replicate using the LunaScript RT SuperMix Kit purchased from New England Biolabs. Quantitative PCR was carried out using 1 µL of cDNA template in a 12 µL reaction volume using Luna Universal qPCR Master Mix purchased from New England Biolabs following the manufacturer's instructions. Each reaction was run for 40 cycles on the qPCR machine listed above. The following parameters were used: 95°C denaturation for 10 seconds, followed by combined annealing and extension at 60°C for 30 seconds. For each pooled embryo sample, RT-qPCR was performed in triplicate. Relative expression levels were normalized to the reference gene *elongation factor 1 alpha (elfa)* calculted using the $2^{-\Delta\Delta Cq}$ method. Raw data can be found in Supplementary Table 5. Primer sequences can be found in Supplementary Table 7.

Supplemental Results

Table S1 Plasmid Information. The name, description, and link to a benchling file of each plasmid used

Plasmid	Description	Benchling Link
pSEE006	dmrt2apromoter driving PTA expression, *U6-1* promoter driving expression of *gata5* sgRNA-1. Insertion region is flanked by Tol2 inverted terminal repeats.	https://benchling.com/s/seq-g5x0ez9AwU9KORpZ0SO0?m=slm-wIB3pRKIhY9MNm1dxvbP
pSEE007	dmrt2apromoter driving PTA expression, *U6-1* promoter driving expression of *gata5* sgRNA-2. Insertion region is flanked by Tol2 inverted terminal repeats.	https://benchling.com/s/seq-BH6FB1J6uv9x0c4lU5ou?m=slm-EznS8PRA3bWOM7dgqIMq
pSEE008	dmrt2apromoter driving PTA expression, *U6-1* promoter driving expression of *gata5* sgRNA-3. Insertion region is flanked by Tol2 inverted terminal repeats.	https://benchling.com/s/seq-lg6BbQquALmsf2lxgCF4?m=slm-vNoVH2ORLlOtVlEhM0hd
pSEE009	dmrt2apromoter driving PTA expression, *U6-1* promoter driving expression of *gata5* sgRNA-4. Insertion region is flanked by Tol2 inverted terminal repeats.	https://benchling.com/s/seq-Wh1frPSZE6CEh2MdIr4Z?m=slm-SA9E0SoKeLSF5dCzLBym
pSEE016	dmrt2apromoter driving PTA expression, *U6-1* promoter driving expression of *ern1* sgRNA-1. Insertion region is flanked by Tol2 inverted terminal repeats.	https://benchling.com/s/seq-QaolYlRIlBaWDPYyxpZF?m=slm-05eyDYYCxmiR6OPYb6Oc
pSEE017	dmrt2apromoter driving PTA expression, *U6-1* promoter driving expression of *ern1* sgRNA-2. Insertion region is flanked by Tol2 inverted terminal repeats.	https://benchling.com/s/seq-ldZhiJrM2DEdVFsofdoO?m=slm-4hrR6wFFLb6i8yVR0HnX
pSEE018	dmrt2apromoter driving PTA expression, *U6-1* promoter driving expression of *ern1* sgRNA-3. Insertion region is flanked by Tol2 inverted terminal repeats.	https://benchling.com/s/seq-aoy7AGdXi7XXqwvMiXbi?m=slm-ZKsZGe3vE2xBCEIArwY2

Table S1 Plasmid Information. The name, description, and link to a benchling file of each plasmid used

pSEE019	dmrt2apromoter driving PTA expression, *U6-1* promoter driving expression of *ern1* sgRNA-4. Insertion region is flanked by Tol2 inverted terminal repeats.	https://benchling.com/s/seq-PEdpX4dq4juidZt8jsW0?m=slm-F4e64Q6FnLmFsBX4zhNq
pSEE023	dmrt2apromoter driving PTA expression, *U6-1* promoter driving expression of *shha* sgRNA-1. Insertion region is flanked by Tol2 inverted terminal repeats.	https://benchling.com/s/seq-gxjovFkd3IICIVQsng1O?m=slm-QyFNMulF7Yamm0JHkFqM
pSEE024	dmrt2apromoter driving PTA expression, *U6-1* promoter driving expression of *shha* sgRNA-2. Insertion region is flanked by Tol2 inverted terminal repeats.	https://benchling.com/s/seq-Z7IceoKIqSIQOoB80JVR?m=slm-BTC7f72OhFR8biElBYGJ
pSEE025	dmrt2apromoter driving PTA expression, *U6-1* promoter driving expression of *shha* sgRNA-3. Insertion region is flanked by Tol2 inverted terminal repeats.	https://benchling.com/s/seq-yfO5HeQ44JMjFSTq5Usw?m=slm-LgsQFWR3JprhZN5HY4yu
pSEE026	dmrt2apromoter driving PTA expression, *U6-1* promoter driving expression of *shha* sgRNA-4. Insertion region is flanked by Tol2 inverted terminal repeats.	https://benchling.com/s/seq-y5G1EmklPh3A19Sflk1F?m=slm-j9X12SfRrHhUnd56fspK
pSEE028	dmrt2apromoter driving PTA expression, *U6-1* promoter driving expression of random sgRNA-1. Insertion region is flanked by Tol2 inverted terminal repeats.	https://benchling.com/s/seq-8wVFZewXvYMttFUO2Llv?m=slm-e98tm0TqdYsVyTm4rN8T
pSEE029	dmrt2apromoter driving PTA expression, *U6-1* promoter driving expression of random sgRNA-2. Insertion region is flanked by Tol2 inverted terminal repeats.	https://benchling.com/s/seq-7umys0ehc8vK1pRtwIco?m=slm-GtpVD0GmF7896rhl6vYh
pSEE030	dmrt2apromoter driving PTA expression, *U6-1* promoter driving expression of random sgRNA-3. Insertion region is flanked by Tol2 inverted terminal repeats.	https://benchling.com/s/seq-9wE2cGpqKnXLUBjyLUsF?m=slm-f8O1Ki1GxRw00MWOo3vu
pNKJ033	dmrt2apromoter driving PTA expression, alpha crystalin promoter driving expression of mCerulean as a selection marker. Insertion region is flanked by Tol2 inverted terminal repeats.	https://benchling.com/s/seq-haa5DspNTJRNc3HarTcw?m=slm-VgjTXUbf7TtwkBHZlu6K
pT3TS-Tol2	Tol2 transposase encoding plasmid	https://benchling.com/s/seq-UOkIz0oHsw728HkMtlyA?m=slm-GC6j4jb30xc3NOJkRvNR
pNJ_009	dmrt2apromoter driving PTA expression, *U6-2* promoter driving expression of *gata5* sgRNA-4. Insertion region is flanked by Tol2 inverted terminal repeats.	https://benchling.com/s/seq-uHXWthrgYokzq6jn0uWu?m=slm-34JbBFOfWLH7yX9GclWr

Table S2 sgRNA Information. The name and sequence of each sgRNA used to home the PTA to their corresponding gene, including the chromosome, gene ID of the target gene, and genomic index of the binding site. Indexes were generated using the NCBI Gene search tool using the NCBI taxonomy ID 7955

sgRNA	Gene ID	Chromosome	Index	Sequence
shha_1	30269	7	40883598-40883617	AGCCTCTAACCTGAACAACT
shha_2	30269	7	40883625-40883644	CAACAAGTGATCAATCTGAC
shha_3	30269	7	40883822-40883841	GTTCATTAGCGGGTCACACA
shha_4	30269	7	40883875-40883894	CAGCCTCACACAGACAGCAG

Table S2 sgRNA Information. The name and sequence of each sgRNA used to home the PTA to their corresponding gene, including the chromosome, gene ID of the target gene, and genomic index of the binding site. Indexes were generated using the NCBI Gene search tool using the NCBI taxonomy ID 7955

sgRNA	Gene ID	Chromosome	Index	Sequence
gata5_1	30482	23	7379337-7379356	ACGTTCTCGCACAGACAGCG
gata5_2	30482	23	7379540-7379559	CAGGACAATTGCATTGACCG
gata5_3	30482	23	7379674-7379693	GGGATAAACTAGATGGTGTG
gata5_4	30482	23	7379711-7379730	TCCAAAGGTAGGACCCATGG
ern1_1	553551	3	55478668-55478687	TGCTCACATAACATTGAAGG
ern1_2	553551	3	55478841-55478860	TGGCGACCCCAAATTAATAA
ern1_3	553551	3	55478884-55478903	TCCAGCAAGTTTTTACACAG
ern1_4	553551	3	55479022-55479041	GCGGTACAGGTGAATTGGGT

Table S3 mRNA Injections Quantitative Survivorship Data. Percent surviving embryos for each cross at 0, 12, 36, and 72 hours post fertilization (HPF)

HPF	gata5	ern	shha	wif	act	Phenol Control	No Injection
0	100	100	100	100	100	100	100
12	18	22	50	89	91	100	94
36	0	17	50	89	91	82	94
48	0	0	0	89	87	82	94
72	0	0	0	89	87	82	94

Table S4 mRNA Injections Quantitative Development Data. Percent normally developing embryos for each cross at 0, 12, 36, and 72 hours post fertilization (HPF)

HPF	gata5	ern	shha	wif	act	Phenol Control	No Injection
0	100	100	100	100	100	100	100
12	0	0	0	50	73	100	94
36	0	0	0	50	73	82	94
48	0	0	0	50	73	82	94
72	0	0	0	50	73	82	94

Table S5 RT-qPCR Quantitative Results of *gata5* Expression Raw Cq values for each biological and technical replicate. Data was analyzed using the $\Delta\Delta$Cq method

Treatment	Target	Time Point (HPF)	Biological Replicate	Cq Value Rep 1	Cq Value Rep 2	Cq Value Rep 3
Control	*elfa*	12	1	14.01	13.84	13.93
Control	*elfa*	12	2	13.22	13.29	13.78
Control	*elfa*	12	3	13.61	13.74	13.58
Experimental	*elfa*	12	1	13.44	13.13	13.08
Experimental	*elfa*	12	2	13.85	14.08	14.20
Experimental	*elfa*	12	3	12.53	12.82	12.61
Control	*elfa*	15	1	11.48	11.12	11.71
Control	*elfa*	15	2	10.29	12.09	12.02
Control	*elfa*	15	3	14.22	13.80	14.01
Experimental	*elfa*	15	1	14.82	14.44	14.60
Experimental	*elfa*	15	2	15.11	14.74	14.69
Experimental	*elfa*	15	3	14.29	13.91	14.07
Control	*gata5*	12	1	21.88	22.27	21.90
Control	*gata5*	12	2	21.86	21.48	21.69

Treatment	Target	Time Point (HPF)	Biological Replicate	Cq Value Rep 1	Cq Value Rep 2	Cq Value Rep 3
Control	*gata5*	12	3	22.17	21.76	22.29
Experimental	*gata5*	12	1	20.65	20.66	20.33
Experimental	*gata5*	12	2	21.28	21.16	21.28
Experimental	*gata5*	12	3	20.25	19.96	20.20
Control	*gata5*	15	1	20.53	20.63	20.36
Control	*gata5*	15	2	20.97	20.97	21.14
Control	*gata5*	12	3	22.40	22.86	22.43
Experimental	*gata5*	15	1	22.13	22.15	21.74
Experimental	*gata5*	15	2	22.02	22.15	22.17
Experimental	*gata5*	15	3	21.46	21.63	21.52

Table S6 RT-qPCR Quantitative Results PTA and *gata5* sgRNA-2 Expression. Raw Cq values for each technical replicate. Data was analyzed using the $\Delta\Delta$Cq method. A "+" in the Reverse Transcriptase column indicates that a reverse transcriptase was included in the reaction. A "-" indicates that a reverse transcriptase was absent

Sample	Target	Reverse Transcriptase	Cq Value Rep 1	Cq Value Rep 2	Cq Value Rep 3
PTA-*gata5* sgRNA-2	PTA	+	29.11	28.81	29.03
wild-type	PTA	+	33.42	37.75	35.98
PTA-*gata5* sgRNA-2	PTA	-	32.06	33.78	33.27
wild-type	PTA	-	34.21	35.15	32.27
PTA-*gata5* sgRNA-2	*gata5* sgRNA-2	+	19.79	19.80	19.95
wild-type	*gata5* sgRNA-2	+	34.40	34.33	34.16
PTA-*gata5* sgRNA-2	*gata5* sgRNA-2	-	34.92	35.18	33.43
wild-type	*gata5* sgRNA-2	-	35.00	38.95	33.45
PTA-*gata5* sgRNA-2	β-*actin*	+	14.50	14.50	14.42
wild-type	β-*actin*	+	16.28	16.25	16.55
PTA-*gata5* sgRNA-2	β-*actin*	-	31.05	30.93	31.26
wild-type	β-*actin*	-	33.22	30.99	33.26

Table S7 RT-qPCR Primer Information. The name, description, and sequence of each primer used in RT-qPCR

Primer	Sequence	Description
oLPP033F	GGACAGTCTTCACGAGCACA	Forward primer that binds to the cDNA of the PTA.
oLPP033R	TTCTCGGGCTTATGCCTTCC	Reverse primer that binds to the cDNA of the PTA.
oLPP044F	CAGGACAATTGCATTGACCG	Forward primer that binds to the cDNA of the *gata5* sgRNA-2.
oAS380	CCAAGTTGATAACGGACTAGCC	Reverse primer that binds to the cDNA of the *gata5* sgRNA-2.
oLPP043F	CGAGCTGTCTTCCCATCCA	Forward primer that binds to the cDNA of β-*actin*.
oLPP043R	TCACCAACGTAGCTGTCTTTCTG	Reverse primer that binds to the cDNA of β-*actin*.

Primer	Sequence	Description
NJ-194	GTCTCGGTCACACAGGCATC	Forward primer that binds to the cDNA of *gata5*.
NJ-195	CTCGGATCACGCTTGAGACA	Reverse primer that binds to the cDNA of *gata5*.
NJ-239	CTTCTCAGGCTGACTGTGC	Forward primer that binds to the cDNA of *elfa*.
NJ-240	CCGCTAGCATTACCCTCC	Reverse primer that binds to the cDNA of *elfa*.

Table S8 Hybrid Cross Quantitative Data. Survival data of the offspring of crossing a homozygous PTA fish and *gata5* sgRNA-2 fish, a homozygous PTA fish and a wild type fish, and two wild type fish. Two crosses were performed per mating pair with 100 embryos per cross (n=200)

Cross	Day 0	Day 1	Day 2	Day 3	Day 4	Day 5	Day 6	Day 7
PTA x *gata5* sgRNA-2	200	120	120	103	103	103	101	97
PTA x Wild Type	200	161	161	161	154	154	154	154
Wild Type	200	165	165	160	158	156	156	151

Table S9 Gonadosomatic Index Quantitative Data. Gonadosomatic index (GSI) and body measurements for PTAgata5-2 x WT and WT x WT crosses

Genotype	Fish ID	GSI	Sex	Total Body Weight (mg)	Gonad Weight (mg)
PTAgata5-2 x WT	1	1.284338209	M	280.3	3.6
PTAgata5-2 x WT	2	0.683060109	M	439.2	3
PTAgata5-2 x WT	3	1.243093923	M	362	4.5
PTAgata5-2 x WT	4	0.256739409	M	389.5	1
PTAgata5-2 x WT	17	15.430132	F	659.1	101.7
PTAgata5-2 x WT	18	17.55975629	F	640.1	112.4
PTAgata5-2 x WT	7	0.295857988	F	608.4	1.8
PTAgata5-2 x WT	8	7.03125	F	409.6	28.8
WT x WT	9	0.737100737	M	447.7	3.3
WT x WT	10	1.563439567	M	831.5	13
WT x WT	11	0.294717751	M	441.1	1.3
WT x WT	12	0.790930662	M	379.3	3
WT x WT	13	13.49892009	F	833.4	112.5
WT x WT	14	6.40625	F	896	57.4
WT x WT	15	7.594322455	F	824.3	62.6
WT x WT	16	9.403623041	F	491.3	46.2

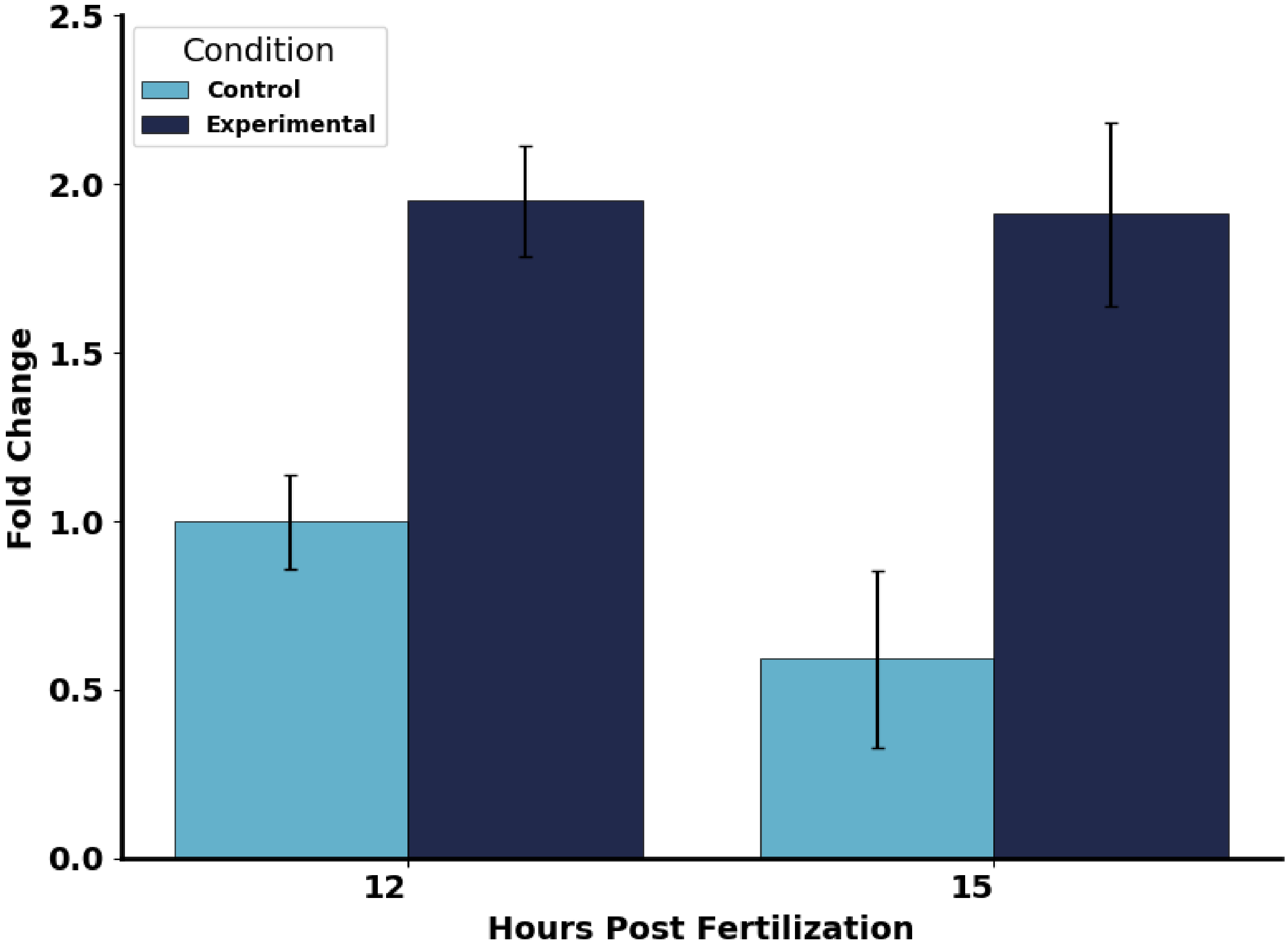

Fig. S1 RT-qPCR Analysis of *gata5* Expression. Fold change in expression of *gata5* relative to elongation factor 1 alpha (*elfa*), 12 and 15 hours post fertilization and injection with the pSEE007 plasmid. Data was calculated using the △△Cq method. Bars represent the mean of 3 biological replicates (n=3)

Test Cross Meta Data.png

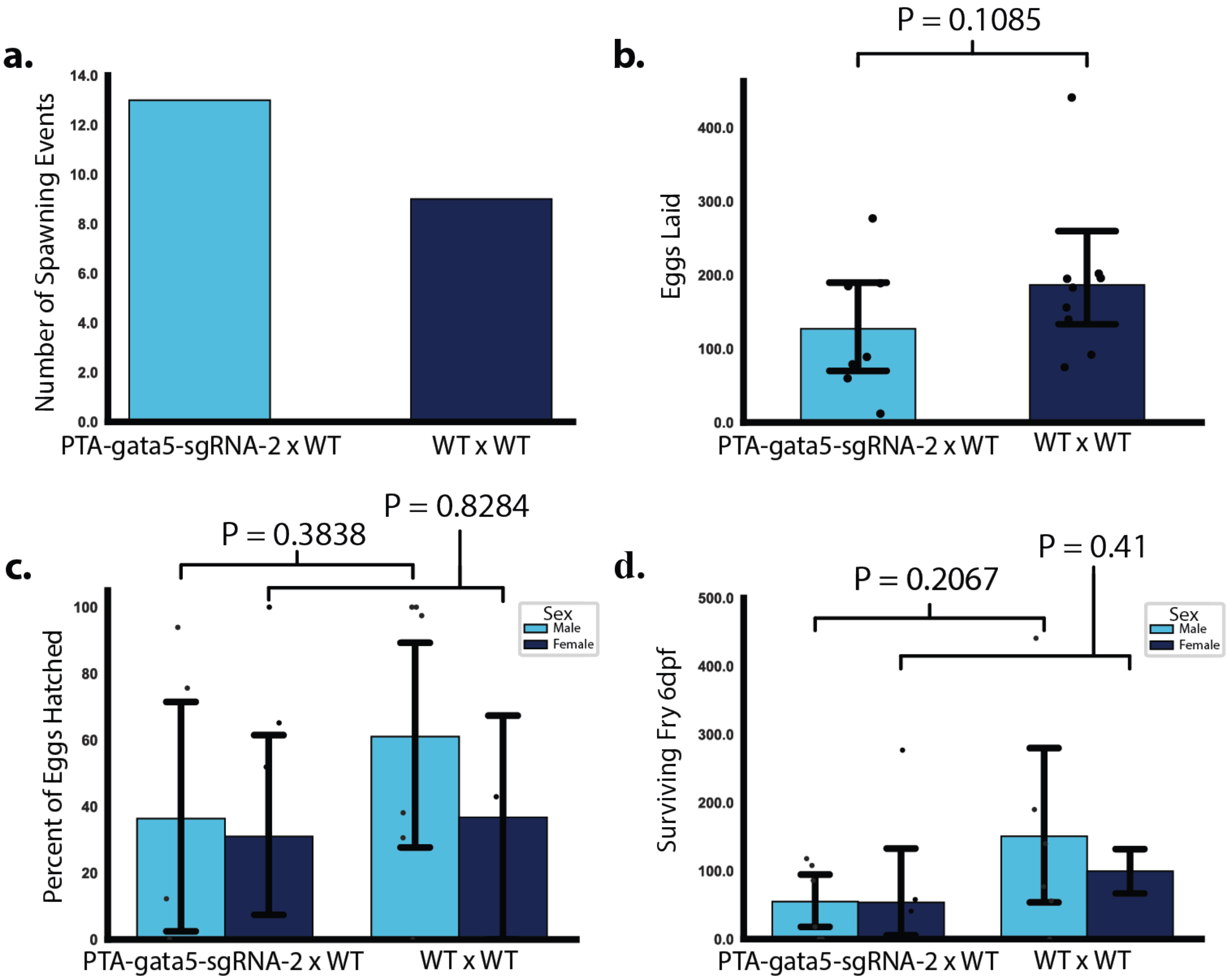

Fig. S2 Reproductive Performance Metrics of PTA-*gata5* sgRNA-2 Offspring. **(a)** The number of spawning events of the zebrafish containing the PTA and *gata5* sgRNA-2 compared to wild-type. **(b)** The number of eggs laid by each female at each spawning event. **(c)** The percent of eggs hatched from each spawning event. **(d)** The number of surviving fry 6 days post fertlization for each spawning event. Statistical significance was determined by a Welch's t-test
